# Variable-order sequence modeling improves bacterial strain discrimination for Ion Torrent DNA reads

**DOI:** 10.1186/s12859-017-1710-0

**Published:** 2017-06-12

**Authors:** Thomas M. Poulsen, Martin Frith

**Affiliations:** 1 Artificial Intelligence Research Center, National Institute of Advanced Industrial Science and Technology (AIST), 2-3-26 Aomi, Koto-ku, Tokyo, 135-0064 Japan; 2 Department of Computational Biology and Medical Sciences, University of Tokyo, Kashiwa, 277-8562 Japan; 3 AIST-Waseda CBBD-OIL, Tokyo, 169-8555 Japan

**Keywords:** Sequence alignment, Higher order, HMM, Ion Torrent, Pathogen detection

## Abstract

**Background:**

Genome sequencing provides a powerful tool for pathogen detection and can help resolve outbreaks that pose public safety and health risks. Mapping of DNA reads to genomes plays a fundamental role in this approach, where accurate alignment and classification of sequencing data is crucial. Standard mapping methods crudely treat bases as independent from their neighbors. Accuracy might be improved by using higher order paired hidden Markov models (HMMs), which model neighbor effects, but introduce design and implementation issues that have typically made them impractical for read mapping applications. We present a variable-order paired HMM that we term VarHMM, which addresses central issues involved with higher order modeling for sequence alignment.

**Results:**

Compared with existing alignment methods, VarHMM is able to model higher order distributions and quantify alignment probabilities with greater detail and accuracy. In a series of comparison tests, in which Ion Torrent sequenced DNA was mapped to similar bacterial strains, VarHMM consistently provided better strain discrimination than any of the other alignment methods that we compared with.

**Conclusions:**

Our results demonstrate the advantages of higher ordered probability distribution modeling and also suggest that further development of such models would benefit read mapping in a range of other applications as well.

**Electronic supplementary material:**

The online version of this article (doi:10.1186/s12859-017-1710-0) contains supplementary material, which is available to authorized users.

## Background

Accurate and fast identification of pathogens is a crucial task for public safety and health [1]. Genomic sequencing provides a high resolution methodology for epidemiological studies and has recently been applied in cases such as cholera outbreaks in Haiti and *Escherichia coli* spread in Germany [2]. One approach in such investigations is to use ‘naive mapping’, where alignment software is used to classify clinical and metagenomic sequenced reads to determine the strain involved (see for example Francis et al. [3]). More recently, frameworks have been developed that incorporate read mapping to classify sequencing data to the correct strain. Such frameworks typically align reads to the entire reference [3–5], whereafter ambiguous alignments are for example reassigned using the joint probabilities of the mapped reads, or use a genomic marker approach such that reads are aligned to a reference of genes [6]. Sequence alignment is hence critical in both the naive mapping approach and classification frameworks, which by default typically utilize software such as BLAST [7] or Bowtie2 [8] but also allow the use of other alignment methods.

Many alignment methods use finite state automa (FSA, [7–10]), which provide an efficient and powerful means of finding the optimal alignment between two sequences. The chance of a match, deletion, or insertion in an FSA is assigned a ‘score’, where a score can be interpreted as a log-likelihood ratio for each of these events. It is hence important to utilize scores that accurately represent the true event probability distributions. Although it is known that higher order genome nucleotide distributions contain unique information, and vary significantly across different types of organisms (see for example [11]), standard FSAs are limited to 0th order likelihood representations that are often approximated using integer values.

Efforts have been made to develop FSAs that use higher order likelihoods, and Crooks et al. [12] for example extended the Smith-Waterman algorithm [13] for dipeptide protein alignment. Their model uses a pairwise substitution score matrix that contains approximated log-likelihood values for dipeptide combinations at different distances apart (i.e. residues separating them), while indels are handled using standard gap alignment penalty scores. No significant improvement was observed compared with the standard Smith-Waterman algorithm however. Hara et al. [14] extended this model to include estimated transition probabilities between dipeptide alignments, and improved protein alignment performance when they modified multiple sequence aligner TCoffee [15] with their method. More indirect methods have also been developed, where Lu and Sze [16] applied a post-alignment algorithm that re-calculates alignment scores by using an average across a position and its neighboring sites. Their method was applied to different multiple sequence aligners and significantly improved protein alignment performance.

Although extended FSAs have shown promising improvements, they have primarily been designed for multiple protein sequence alignment and do not model probability distributions directly. They are also limited to dipeptides or post-alignment processing, and use standard gap penalties for indels. Thus for nucleotide sequence alignment and classification, a different model may be more suitable.

Paired hidden Markov models (HMMs) are very similar to FSAs but use probabilities instead of scores, and are thus well suited if we want to directly model the probability distributions for different alignment events. Higher order HMMs in particular, can provide a more detailed representation of alignment distributions than FSAs and 0th order HMMs, but their application in sequence alignment has so far been very limited. This is partly because paired HMMs can be slow, but also due to additional complexities that are introduced by higher order models.

Attempts to move beyond a standard 0th order paired HMM for sequence alignment has hence been more limited than the case of FSAs, though Nánási et al. [17] recently developed two different types of HMMs that are able to use previous sequence information to process tandem repeats. Both of their models use a standard paired HMM, but in their first model they add profile HMM states to handle repeats, while in their second model they extend the standard model with states that follow the implementation in the repeat finder TANTAN [18]; an HMM developed for detecting repeats in single nucleotide/protein sequences. Nánási et al. do not model probability distributions, but instead utilize estimated indel and mutation rates based on earlier studies. Their models have so far only been tested in simulated scenarios but show a promising approach for scoring repeat regions differently than other parts of a sequence.

In addition to the Nánási et al. [17] models, HMMs have been developed that correct alignments of homopolymer regions when using Ion Torrent reads [19, 20]. Such methods do not utilize higher order models to capture general matches and indels, but specifically model repeats of different lengths. As these approaches correct existing alignments, they are complementary to read alignment and can further improve results in the case of homopolymer sequencing errors.

Although it is common to use sequence alignment when performing pathogen classification with sequenced DNA, alternative approaches have been developed for classifying guanosine triphosphate proteins (GTPases) and protein folds, which use techniques such as neural networks [21], random forests [22], and profile-based composition vectors [23]. Such techniques rely on at least one descriptor such as hydrophobicity or secondary structure, and for example perform linear interporlation to normalize sequence lengths, so it is unclear how well such models can be applied in the case of classification based on DNA reads. Their use of sequences and data dimension reduction offer an interesting approach however, and may provide benefits if adopted for next generation sequencing and classification.

We here present a variable order paired HMM for sequence alignment and read classification that we term VarHMM, which addresses higher order modeling issues and exploits similarities between reads to improve processing time. In contrast to previous methods, we directly model probability distributions for higher order alignment and indel likelihoods, and our method has been developed for nucleotide sequence alignment and classification. VarHMM uses the same states as a standard paired HMM, and is not mutually exclusive with HMMs such as the Nánási et al. [17] models, and can for example be extended with states to handle tandem repeats. Our model is applicable for distinguishing amongst known bacterial strains, and is evaluated in classification tests where it is compared with commonly used alignment software packages as described in the Results section. Several different next generation sequencing methods can be utilized for pathogen classification, and we focused on reads from Ion Torrent sequencing which offers a fast and viable method for pathogen detection [24].

## Implementation

### Sequence data and aligners

Our alignment method VarHMM, is based on a higher order paired HMM implementation. We implemented VarHMM as well as 0th, 1st, and 2nd order models for comparison (HMM0, HMM1, and HMM2 respectively). In the following, we describe how seeding, alignment, and training are performed, and how VarHMM and the higher order paired HMMs were implemented.

### Sequence alignment using paired HMMs

In this section, we briefly describe how the probability of a single alignment is calculated, and in the next section we will explain how this can be used to find the ‘best’ alignment.

**Fig. 1:**
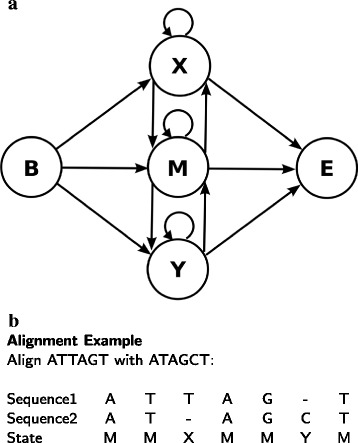
Paired HMM: **a** States and connections: B = begin and E = end states, and M = match, X,Y = output states (B to E connection not shown). **b** Example of an alignment between two sequences (state that was entered in the paired HMM is shown for each position of the alignment)

All of the paired HMMs that we implemented use five states as shown in Fig. 1 a, where for a given alignment, we start with the Begin state (B) and an finish with the End state (E). We enter a match state (M) when two nucleotides align, and enter an output state (X or Y) when there is a nucleotide output in only one of the sequences as can be seen in the example in Fig. 1 b.

If we use a 0th order paired HMM to align Sequence1 and Sequence2 in Fig. 1 b, then the probabilty of their alignment *p*_0_(*S*1:*S*2) is:

$$\begin{array}{*{20}l}{} &p_{0}(S1:S2) \,=\, a_{BM}\! \cdot\! p(A\!:\!A)\! \cdot\! a_{MM}\! \cdot p(T\!:\!T)\!\cdot\! a_{MX}\! \cdot\! q_{x}(T)\\ {}&\cdot\! a_{XM} \!\cdot\! p(A\!:\!A)\! \cdot\! a_{MM} \!\cdot\! p(G\!:\!G)\! \cdot\! a_{MY} \cdot q_{y}(C) \cdot a_{YM} \cdot p(T:T)\\ {}&\cdot a_{ME} \end{array} $$

where the *a* values denote the state transion probabilities (e.g. *a*_*MX*_ is the probability of transitioning from M to X), and for example *p*(*A*:*A*) is the probability that A matches with A and *q*_*x*_(*T*) is the probability of outputting a T nucleotide in Sequence1 (all of these probabilities are derived by training a paired HMM which we will describe in the Training section).

In the case of a 1st order model, the alignment probability of Sequence1 and Sequence2 is instead:

$$\begin{array}{*{20}l}{} &p_{1}(S1:S2)=a_{BM}\cdot p(A:A)\cdot a_{MM}\cdot p(T:T|A:A)\cdot a_{MX}\\ {}&\cdot q_{x}(T|T) \cdot a_{XM} \cdot p(A:A) \cdot a_{MM} \cdot p(G:G|A:A)\cdot a_{MY}\\ {}&\cdot q_{y}(C|G) \cdot a_{YM}\cdot p(T:T) \cdot a_{ME} \end{array} $$

where *p*(*T*:*T*|*A*:*A*) for example is the probability that T matches T given the prior alignment between A and A, and *q*_*y*_(*C*|*G*) is the probability of outputting a C with a G occurring immediately before in Sequence2. When a gap occurs immediately before a match between two nucleotides (or a gap occurs immediately prior in the sequence where there is a nucleotide output), then a 0th order model is used to calculate the probability as can be seen in the case of the last match which uses *p*(*T*:*T*). As will be described in further detail in the next section, all of the paired HMM models use the highest order possible after previous gaps in the alignment have been taken into account, while VarHMM *also* adjusts the order based on which probabilities have sufficient training data to be considered reliable.

### Viterbi and higher order paired HMMs

In order to find what we think is the best alignment between two sequences, we compare the probabilities of different alignments. The general idea is to store the different alignment probabilities in an *alignment matrix* and then use them to find the alignment we think is best. For example, we can use the alignment matrices to store the probability that we align the first nucleotide in Sequence1 in Fig. 1 b to nucleotide 1,2,...,6 in Sequence2, and then repeat again for Sequence1 nucleotides 2–6.

**Fig. 2:**
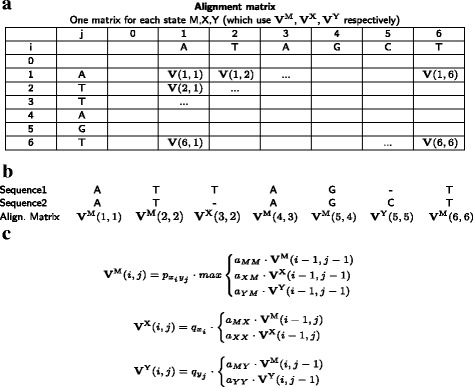
Finding the alignment using Viterbi: **a** For each state M, X, Y we calculate an alignment matrix: each matrix position contains the alignment probability **V**(*i,j*) up until the position *i* in Sequence1 and *j* in Sequence2 (shown in Fig. 1). **b** Example of how the alignment matrices are traversed using the alignment from Fig. 1. **c** To find the alignment with the highest probability: **V**(*i,j*) is calculated for each of the states M: **V** ^M^(*i,j*), X: **V** ^X^(*i,j*), Y: **V** ^Y^(*i,j*). The probability of transitioning between two states is given by *a* (e.g. from X to M is *a*_*XM*_), and is multiplied by the alignment probability *V* of the state and position we are coming from (for example **V** ^M^(*i,j*)=*a*_*XM*_·**V** ^X^(*i*−1,*j*−1) if going from state X to M). We then transition from the previous state and position which maximizes **V** ^M^(*i,j*) for alignment matrix M, and **V** ^X^(*i,j*), **V** ^Y^(*i,j*) for the X,Y matrices. We perform this for each *i,j* position and always store which previous state was transitioned from. The alignment with the highest probability *V*_*max*_ can then be found using traceback: given a Sequence1 of length *n* and a Sequence2 of length *m*, traceback starts from the state given by: *V*_*max*_=*max*{*a*_*ME*_·**V** ^M^(*n,m*), X: *a*_*XE*_·**V** ^X^(*n,m*), Y: *a*_*YE*_·**V** ^Y^(*n,m*)} where *a*_*ME*_, *a*_*XE*_, and *a*_*YE*_ are the transition probabilities from M, X, and Y respectively to E

There are different ways to implement the alignment matrices and select a final alignment, but one approach is to use the Viterbi algorithm [25], which uses the matrices to find the path that gives us the highest alignment probability. Figure 2 provides a short summary of the Viterbi algorithm and how it is implemented with a paired HMM (for a more in depth description of paired HMMs and use with the Viterbi algorithm see for example Durbin et al. [26]), where Fig. 2 a shows an example of an alignment matrix, Fig. 2 b illustrates how the alignment matrices are traversed for Sequence1 and Sequence2, and Fig. 2 c describes how the alignment with the highest probability is found using Viterbi.

One difficulty with higher order paired HMMs is that they need to look back at the preceding nucleotides at each position in an alignment. HMMs are designed with the assumption that probabilities in a state are independent however, and so using higher ordered probabilities with a paired HMM and an algorithm such as Viterbi introduces difficulties that must be addressed. In particular, a higher order probability is dependent on the preceding alignment, but matches between the preceding nucleotides are not decided until we have calculated the alignment matrices as seen in Fig. 2. For example, if there are indels in the preceding aligned nucleotides then we must take them into account when we calculate the higher order probabilities at each position in an alignment matrix. To address this issue, we exploited the same traceback system as the Viterbi algorithm to keep track of when an insertion was last made for each position in an alignment. In the following, we consider the alignment between Sequence1 and Sequence2 to describe how this functions, and use *i* and *j* to refer to the nucleotide positions in Sequence1 and Sequence2 respectively.

In the higher order models, the Markov order that is used for the alignment probability is dependent on what occurred in the preceding states, which can be seen if we consider the three following examples:

**Table:** 

1)	Sequence1	T	A	A	T	C	G
	Sequence2	T	A	A	T	-	G
	States	M	M	M	M	X	M

2)	Sequence1	T	A	A	T	C	G
	Sequence2	T	A	A	T	-	-
	States	M	M	M	M	X	X

3)	Sequence1	T	A	A	T	-	-
	Sequence2	T	A	A	T	C	G
	States	M	M	M	M	Y	Y

If we are in the M state, and the previous state produces an indel as shown in the first example, then we utilize an order of 0 because the immediate position before our current one contains a gap in Sequence2. In contrast, if we are in state X as in the first and second examples, then we only need to be concerned with preceding gaps in Sequence1 and can ignore Sequence2 (while we only need to consider preceding gaps in Sequence2 when in state Y as in the third example). At each position *i,j* in the alignment matrix (Fig. 2), we therefore count the number of nucleotides since a gap last occurred in either sequence, which we store in matrices **X**_c_ and **Y**_c_: **X**_c_ counts the number of nucleotides since a gap was made in Sequence1 and **Y**_c_ contains the counts for Sequence2.

**Fig. 3:**
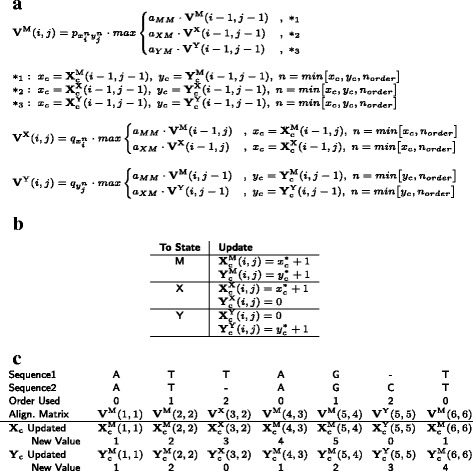
**a** The Viterbi equations, extended for the higher order paired HMMs. In addition to the **X**_c_ and **Y**_c_ counts, a higher order HMM is also restricted by its order which we term *n*_*order*_ (see Fig. 2 for definition of other variables). The order used to calculate $p_{x^{n}_{i}y^{n}_{j}}$, $q_{x^{n}_{i}}$, $q_{y^{n}_{j}}$ is then taken as the minimum between the *X*_*c*_,*Y*_*c*_ counts and *n*_*order*_ as shown above. **b** Update of count matrices, where $x^{*}_{c}$ and $y^{*}_{c}$ are the *x*_*c*_ and *y*_*c*_ values we from the Viterbi path we take in the (**a**) equations (see text for details) **c** The order used and the **X**_c_ and **Y**_c_ values updated at each alignment position for a 2nd order paired HMM (using Fig. 2 example). The order used at each position is based on the **X**_c_ and **Y**_c_ counts from the *previous* position and the equations in (a). For example when A aligns to A at **V** ^M^(3,4), the previous alignment position was at (2,3) in state X, and so the order used to calculate **V** ^M^(3,4) is the minimum of [$\mathbf {X}^{\mathrm {X}}_{\mathrm {c}}(2,3)$, $\mathbf {Y}^{\mathrm {X}}_{\mathrm {c}}(2,3)$, *n*_*order*_], which is equal to zero as $\mathbf {Y}^{\mathrm {X}}_{\mathrm {c}}(2,3)=0$

As shown in Fig. 3 a and b, the counts are updated at each position *i,j* similar to how the Viterbi matrices are updated (where the state column in Fig. 3 b refers to the state that we are in at position *i,j*). If we for example enter a match state, then the number of times that we had a nucleotide output in both Sequence1 and Sequence2 increases by one, and we therefore increase the $\mathbf {X}^{\mathrm {M}}_{\mathrm {c}}(i,j)$ and $\mathbf {Y}^{\mathrm {M}}_{\mathrm {c}}(i,j)$ counts by one. This is done by adding a value of one to $x^{*}_{c}$ and $y^{*}_{c}$, which represent the *x*_*c*_ and *y*_*c*_ values that are chosen from the Viterbi path when we use the equations in Fig. 3 b (i.e. $x^{*}_{c}$ and $y^{*}_{c}$ give us the counts from the state we are coming from in the Viterbi path). In contrast, if we entered state X, then there was a nucleotide output in Sequence1 and a gap in Sequence2. In this case, we increase the counts for $\mathbf {X}^{\mathrm {X}}_{\mathrm {c}}(i,j)$ by one, but set $\mathbf {Y}^{\mathrm {X}}_{\mathrm {c}}(i,j) = 0$ (and vice versa for state Y, where there is an output in Sequence2 and gap in Sequence1, we increase the counts for $\mathbf {Y}^{\mathrm {X}}_{\mathrm {c}}(i,j)$ by one and set $\mathbf {X}^{\mathrm {X}}_{\mathrm {c}}(i,j)= 0$). We again update using the counts from the previous state in the Viterbi path, as given by $x^{*}_{c}$ and $y^{*}_{c}$. For initialization at position *i*=0 and *j*=0 all counts are set to zero.

The **X**_c_ and **Y**_c_ counts are then used to help determine the order when we calculate the probability of an alignment $p_{x^{n}_{i}y^{n}_{j}}$ and the nucleotide output $q_{x^{n}_{i}}$, $q_{y^{n}_{j}}$ at each *i,j* position for the alignment matrices as shown in Fig. 3 a and c.

### Datasets

We used sequencing data from *E. coli K12* substrain *DH10B* for training and testing. Two different sequencing sets from an Ion Torrent PGM 314v2 were used (datasets from https://figshare.com/s/6101b0605da5e9a6f72e): 1) C24-698, mode read length >200bp (average read length 244bp) and 35.7X average coverage, 2) B22-730, mode read length of 400bp (average read length 325bp) and 33.9X average coverage (494921 reads). We refer to the first dataset as R200 and the second dataset as R400. Half of the R200 dataset was used for training and the other half testing, which we term R200-train and R200-test respectively.

### Training

The training set was constructed by first aligning the R200-train reads to *E. coli DH10B* with Last using its default parameters (see Additional file 1: Section S4 for details). Only alignments that were greater than 150 in length and which had a Last mismap probability score less than 0.001 were used for training. We then utilized maximum likelihood estimation (MLE) with this set for all of the pair HMMs to estimate initial transition, alignment, and output probabilities (see for example Durbin et al. [26] section 3.3 for details on how transition, alignment, and output probabilities are calculated from a set of alignments for paired HMM training using maximum likelihood estimation). After initialization, we used the Viterbi algorithm to re-align all of the alignments in the training dataset with the HMM, and re-estimated all of the probabilities with MLE. Training was stopped if the difference between the logarithmic values of the total probability of the new and old alignments was less than *p*_*stop*_=0.8, or if *n*_*it*_=5 iterations was reached (for both the full dataset used in the Results, and the half dataset used in Additional file 1: Section S2.3, training stopped after 5 iterations).

### VarHMM: a variable higher order paired HMM

When training higher order HMMs, the training data set might not be sufficiently large to properly capture the higher order distributions in which case the model may perform worse than a lower order HMM. This was noticeable in the training set, where a 3rd order HMM appeared to yield worse results than a 2nd order model. To address this issue, we implemented VarHMM, which is a variable order paired HMM. VarHMM uses the same implementation as the higher order paired HMMs, but for each order (0th to 3rd order), it also counts the number of samples for all of the alignment and nucleotide output probabilities (e.g *p*(*A*|*A*),*p*(*C*|*A*),...etc.). If the counts for a given alignment or output is below a count of *c*_*min*_, then a lower-order conditional probability is used (highest possible is then selected). We used a maximum order of 3 for both the M and the X and Y states, and a value of 100 for *c*_*min*_.

### Seeding and alignment

VarHMM and the 0th, 1st, and 2nd order paired HMM models all performed global alignment, and used the same method for seeding and aligning sequences. A detailed description of how this was implemented, as well as parameter values used for all of these models is provided in Additional file 1.

## Results and discussion

To evaluate VarHMM and the different paired HMMs, we compared their performance with commonly used alignment software packages: Bowtie2 [8], Last [9], Segemehl [10], and TMAP (part of the Torrent Suite at https://github.com/iontorrent/TS). We evaluated each method by aligning reads from *E. coli K12* substrain *DH10B*, and focused on classification between substrains as this can be particularly difficult due to the high degree of similarity.

Reads from the R200 and R400 datasets were aligned to a sequence database comprising two genomes: *E. coli DH10B* (NC_010473.1) and either 1) *E. coli MG1655* (U00096.3), or 2) *E. coli C3026* (NZ_CP014272.1). All of the alignment software parameters except for Last were set according to Caboche et al. [27] (see Additional file 1 for Last parameters and for version details of all the alignment methods).

In each test, if a read was classified to the *E. coli DH10B* genome it was marked as a correct mapping. If a read was mapped to more than one position it was classified to a genome according to two criteria: 1) If all the aligned positions were in the same genome, then the read was classified to that genome, 2) If the first criterion were not true, then the highest scoring alignment was selected if it was greater than the second best alignment by a given threshold value (which we varied for all the different alignment methods, altering the percent of wrongly and correctly mapped reads; for Last we varied the maximum mismap probability which has a similar effect).

In the case of TMAP, the first criterion caused the results to decrease significantly in one of the test sets because it was unable to find any matches in the *E. coli DH10B* genome for a set of reads (this significant decrease was not observed for any of the other aligners). We therefore derived two sets of results for TMAP, with one set based on both criteria that we label ‘TMAP’ in the plots, and an additional set where only the second criterion was used which we label ‘TMAP*’ (i.e. in ‘TMAP*’ a read is only classified if the difference between the best and second best alignment scores is above the supplied threshold, while in ‘TMAP’ a read is *also* classified if all of the alignments occur in only one of the genomes).

**Fig. 4:**
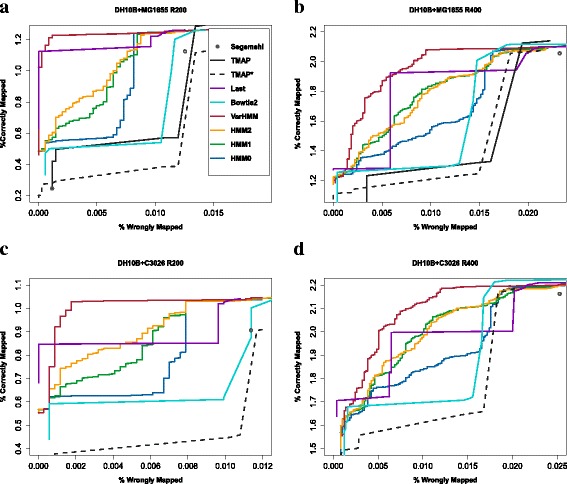
Comparison of different alignment methods: **a** R200 reads aligned to *DH10B* and *MG1655*, **b** R400 reads aligned to *DH10B* and *MG1655*, **c** R200 reads aligned to *DH10B* and *C3026*, **d** R400 reads aligned to *DH10B* and *C3026*

For each alignment method, we compared the percent of wrongly mapped reads with the percent that was correctly mapped. Figure 4 a and b show the results for the first test set, where reads from R200 and R400 where aligned to *E. coli DH10B* and *MG1655*. For both the R200 and R400 sets, VarHMM showed the best performance, followed by Last which at most points outperformed the 0th, 1st, and 2nd order models. The 1st order model sometimes performed better than the 2nd order model, and illustrates how the performance of a higher order paired HMM can decrease due to insufficient training data as described in the Methods section. Bowtie2 performed slightly better than TMAP, while TMAP scored higher than Segemehl for the R400 set in Fig. 4 b but worse for the R200 set in Fig. 4 a.

When using both criteria 1 and 2 for classifying reads, TMAP was able to align a small number of extra reads compared with the other alignment methods when the percent of wrongly mapped reads was relatively high in Fig. 4 a and b. In this case, TMAP appears to only have aligned reads to *DH10B* which the other aligners likely matched to both *DH10B* and *MG1655*. Although this benefited TMAP in the first test, in the second test with *E. coli DH10B* and *C3026*, TMAP aligned a set of reads to only *C3026* and not *DH10B*, resulting in a significant number of incorrectly mapped reads.

Figure 4 c and d show the results for *E. coli DH10B* and *C3026*. For the sake of visual clarity, the results for TMAP are not included but shown in Additional file 1: Figure S1 instead (TMAP started at approximately 0.06 and 0.04% wrongly mapped reads in Fig. 4 c and d respectively, making it harder to distinguish the results of the other alignment methods). The results in Fig. 4 c and d show that VarHMM again performed the best and was followed by Last. Bowtie2 showed stronger results than TMAP*, while the TMAP* results were better than Segemehl in the case of the R400 reads but worse for the R200 dataset. Although the TMAP results in both tests didn’t differ greatly from Segemehl and Bowtie2, TMAP has been designed for Ion Torrent sequencing, and so the results were lower than expected.

As VarHMM was trained using Last alignments, the HMM models may have achieved higher accuracy than the other aligners in part due to Last’s performance. To rule out this possibility, and also ensure that our estimated parameters (Additional file 1: Table S2) for VarHMM were not tailored to the Last training data, we trained two separate versions of VarHMM with alignments from Bowtie and TMAP alignments (see Additional file 1: Section S2.2 for details). A comparison was then made between these two versions of VarHMM and the other alignment methods, where we used the same parameters for VarHMM as before (parameters shown in Additional file 1: Table S2). As can be seen in Additional file 1: Figure S2, this prompted a smaller decrease in VarHMM’s performance compared with the results in Fig. 4, but performance was otherwise similar for both VarHMM trained with Bowtie and with TMAP alignments.

Unlike the other HMMs, VarHMM uses *c*_*min*_ as a cutoff as explained in the “VarHMM: a variable higher order paired HMM” section. This posed questions such as how often the *c*_*min*_ threshold was met during training, and how results would be affected by a smaller training set. An analysis of how frequently the *c*_*min*_ threshold is met, and what portion of the data the higher orders capture, is described in Additional file 1: Section S2.3 (results shown in Additional file 1: Table S3). Interestingly, increasing *c*_*min*_ only caused more rare cases to be excluded in the higher order modeling of the M state, while the X and Y states were largely unaffected. As an additional control, we trained a VarHMM model with half of the Last training data that was used to train the VarHMM in Fig. 4 (using the same *c*_*min*_=100 value as before). The results are shown in Additional file 1: Figure S3, and as can be seen, VarHMM showed only very minor changes compared with Fig. 4, illustrating how the variable order model can compensate for changes in the training data size.

In addition to the alignment tests performed in Fig. 4, we made a more detailed comparison between VarHMM and the other aligners to measure how well they could determine a strain using a given set of reads. We implemented a similar test framework as Francis et al. [3], who used low coverage datasets to model factors such as use of multiplexed sequencing and contamination of samples from other genomic sources (see Additional file 1: Section S2.4 for test details). Similar to the results seen in Fig. 4, VarHMM showed the best performance, followed by Last and then Bowtie and TMAP. The results suggest that an aligner such as Bowtie or Last is likely to provide the same accuracy when classifying a purified sample to a small number of strains, while VarHMM can provide greater accuracy in more difficult scenarios as described in Francis et al. [3]. Our results here also illustrate how VarHMM can be applied for pathogen detection, or for example to distinguish between pathogenic and non-pathogenic strains.

A comparison between the run times of the different alignment methods was made using the R200 dataset with *E. coli DH10B* and *MG1655* and can be seen in Table 1. All of the aligners were run on a basic system consisting of a 1.4 GHz Intel Core i5 and 4 GB of memory to see if they could run on a relatively lower end system. The paired HMMs were the slowest (Table 1 only shows VarHMM results but the other paired HMMs had similar run times), followed by Segemehl. Bowtie2, Last, and TMAP were all significantly faster, though Bowtie2 had the fastest run time. Although VarHMM was able to run on a low level system, using more high end specifications such as an Intel Core i7 based system is hence recommended, allowing for run times under an hour.

**Table 1:** Run times for the different alignment methods for aligning the R200 reads to DH10B and MG1655

Aligner	Run time (minutes)
Bowtie2	1.10
Last	1.85
TMAP	2.10
Segemehl	48.24
VarHMM	210.45

## Conclusions

VarHMM achieved the best performance amongst all of the alignment methods that we compared with. Although we focused on Ion Torrent reads, accurately modeling nucleotide distributions is fundamental to alignment of any sequenced data. As such, a higher order based model such as VarHMM should also provide advantages with other types of sequencing.

All of the paired HMMs were slower than the other alignment methods that we compared with, and future work will be focused on improving algorithm design and implementation to increase speeds. We also limited VarHMM to a maximum 3rd order model, but it would be possible to make a more dynamic design that uses higher orders for the output states to further improve performance.

VarHMM is complementary rather than competitive to fast aligners such as Bowtie2. Its relative slowness makes it inappropriate for huge datasets, and its niche is smaller datasets where best-possible accuracy is desired. An obvious example is bacterial strain discrimination, where our results suggest that VarHMM can provide better sensitivity and accuracy for more difficult scenarios as described in the Results section. In addition to smaller datasets, VarHMM could also be applied alongside a fast alignment method for mapping shorter reads as they tend to be harder to align. Ultimately, standard zero-order methods will hit an accuracy ceiling that can be broken only by higher-order sequence modelling.

Pathogen detection and biosurveillance is crucial for public safety, and accurate classification of reads is fundamental when using epidemiological methodologies. Our results show that VarHMM can achieve both high accuracy and sensitivity when classifying reads, providing a powerful tool that can be used for pathogen detection. These findings also have important implications going forward, where our results demonstrate the advantages of using higher ordered probability distribution modeling, and suggest that further development of such models would benefit read mapping in a range of other applications as well.

## Availability and requirements

**Project name:** VarHMM

**Project home page:** https://github.com/gitvarhmm/varhmm

**Operating system(s):** Windows, Mac, Linux

**Programming language:** C++

**Other requirements:** None

**Licence:** GNU General Public License v3.0

## Additional file

Additional file 1 Supplementary information. This file contains the following sections: S1 - Details about seeding, alignment steps, and parameter selection. S2 - Contains details and graphs for additional testing and results described in the Results section of the main text. S3 - Version details for the different aligners. S4 - Parameters used for Last training and alignment, and also Last training results. S5 - Where to download and how to use Varhmm software, as well as training results from VarHMM. (PDF 318 kb)

## Abbreviations

E. coli: Escherichia coli
FSA: Finite state automaton
HMM: Hidden Markov model
HMM0: 0th order hidden Markov model
HMM1: 1st order hidden Markov model
HMM2: 2nd order hidden Markov model
VarHMM: Variable order hidden Markov model
