# Supplementary material for: Variable-order sequence modeling improves bacterial strain discrimination for Ion Torrent DNA reads
Source: BMC Bioinformatics. 2017 Jun 12;18:299. doi: 10.1186/s12859-017-1710-0 (PMC5469136; doi:10.1186/s12859-017-1710-0)
Supplement: Additional file 1 — Supplementary information. This file contains the following sections: S1 - Details about seeding, alignment steps, and parameter selection. S2 - Contains details and graphs for additional testing and results described in the Results section of the main text. S3 - Version details for the different aligners. S4 - Parameters used for Last training and alignment, and also Last training results. S5 - Where to download and how to use Varhmm software, as well as training results from VarHMM. (PDF 318 kb) [file 12859_2017_1710_MOESM1_ESM.pdf]

Supplementary Text for: Variable-order sequence modeling improves bacterial strain discrimination for Ion Torrent DNA Reads

Thomas M. Poulsen and Martin C. Frith

S1 Methods

S1.1 Seeding

The general idea of our seeding method is to scan through the entire reference and look for short matches using the start of a read and then exploit read similarities to reduce the number of calculations and processing time. These short matches are pre-calculated one time only for a paired HMM once it has been trained. For the pre-calculated values, a paired HMM is used to calculate scores for all possible alignment combinations that are 8 nucleotides in length as shown in table S1. The pre-calculated values are then stored in the following matrix:

$$\mathbf{S} = \begin{pmatrix} \mathbf{S}_{1,1} & \mathbf{S}_{1,2} & \cdots & \mathbf{S}_{1,65536} \\ \mathbf{S}_{2,1} & \mathbf{S}_{2,2} & \cdots & \mathbf{S}_{2,65536} \\ \vdots & \vdots & \ddots & \vdots \\ \mathbf{S}_{65536,1} & \mathbf{S}_{65536,2} & \cdots & \mathbf{S}_{65536,65536} \end{pmatrix} \quad (1)$$

where there are 65536 combinations for a sequence that is 8 nucleotides in length. Each of the two sequences in an alignment is hence given an index value as seen in table S1, where the index value is calculated as the sum: $n_0{\cdot}4^7{+}n_1{\cdot}4^6{+}n_2{\cdot}4^5{+}n_3{\cdot}4^4{+}n_4{\cdot}4^3{+}n_5{\cdot}4^2{+}n_6{\cdot}4^1{+}n_7{\cdot}4^0$, n_1 to n_7 are numerical values that represent the nucleotide at each position in a sequence from position 0 to 7 with: A = 0, C = 1, G = 2, T = 3 (for example AAAAAAAT would give an index value of 3).

Alignment Score	Index Values:	Alignment
$s_{0,0}$	$0, 0$	AAAAAAAA<br>AAAAAAAA
$s_{0,1}$	$0, 1$	AAAAAAAA<br>AAAAAAAC
...	...	
$s_{65536,65536}$	$65536, 65536$	TTTTTTTT<br>TTTTTTTT

Table S1: Examples of 8-length alignments and corresponding alignment scores

The alignment score s is defined as:

$$s = \frac{V_{max}}{L_{read}} \quad (2)$$

where V_{max} is the probability of the highest scoring Viterbi alignment as described in the 'Material and Methods' and Figure 2 of the main text, and L_{read} is the length of the read

and is used for normalization. The above equation for s is used to calculate the score for all paired HMM alignments (for example, for both the above pre-calculated alignments and the alignments described in the next section).

S1.2 Making Alignments and Parameter Selection

Once the S matrix described in the previous section has been calculated, we can proceed to align a given set of reads with any reference. This involves two initialization steps followed by the main process:

Initialization

1. Only align reads that have a length that is longer than a value of L_{min}. Sort all reads alphabetically using the Quicksort algorithm (1).

2. Scan through the reference sequence, and at each position retrieve a segment of the reference that is 8 nucleotides in length. Convert the 8-length segment to an index value k_n (as shown in table S1), where n refers to the position in the reference that k_n was calculated. Each k_n value is stored in a vector of indexes $\mathbf{r} = [k_1, k_2, ..., k_N]$, where given a reference of length L_{ref} then $N = L_{ref} - 8$.

Main Process

1. Compare the first 8 nucleotides of a read with the previous read. If different (or if we are processing the very first read), then proceed to step 2 (otherwise go to step 3).

2. For the first 8 nucleotides of the current read m, calculate the read index value l_m. Scan through the reference and at each position n get the index value $k_n = \mathbf{r}(n)$ and then the alignment score in $\mathbf{S}$. Going through positions 1 to N in the reference we get: $\mathbf{S}(k_1, l_m)$, $\mathbf{S}(k_2, l_m)$, ... $\mathbf{S}(k_N, l_m)$. If a score is greater than s^8_{min}, then the reference position is stored in a vector $\mathbf{u}$ which contains all the positions in the reference where we think the read might align to. In the case we only want to use exact matches, then a comparison is just made between each k_n index from the reference and the read index l_m (in which case the S matrix calculation is optional), and we store a reference position in $\mathbf{u}$ if $k_n = l_m$.

3. For each alignment in $\mathbf{u}$, we make a rough comparison between all of the read and the reference segment to eliminate any obvious mismatching alignments. This is done by counting the number of A, C, G, and T nucleotides in both the read and reference segment for every window of w_{len} nucleotides (if a read and reference section length are less than w_{len} then they are not included as a window and ignored). For each window, we calculate the difference in nucleotide counts between the read and reference (i.e. for each window $|\#\text{As in read} - \#\text{As in reference}| + ... + |\#\text{Ts in read} - \#\text{Ts in reference}|$), and calculate z as the average across all the windows.

4. For each alignment in $\mathbf{u}$ that has a z value that is less than z_{max}, or where the read length is less than w_{len}, we calculate an alignment that is 24 in length (or less if the read is shorter). We then create a new array $\mathbf{v}$ which contains the 24-length alignments that have a score greater than s^{24}_{min}.

5. For each alignment in $\mathbf{v}$, we extend the alignment so that the entire read is aligned and the best and 2nd best alignments are saved (the next read is then processed and we return to step 1). If $\mathbf{v}$ is empty, or if the best score is less than s_{min}, then the first 8 nucleotides of the read are removed and steps 1-4 are repeated (up to a maximum number of N_{rep} times).

Parameter	Value
s_{min}	-2.5
s^{8}_{min}	-2.5
s^{24}_{min}	-3.2
z_{max}	7
w_{len}	40
N_{rep}	5
L_{min}	20
c_{min}	100

Table S2: Values of parameters used in the main process

The values of the constants used in the above steps are shown in table S2. The s^{8}_{min} cutoff was selected so that only exact matches of 8-length were considered for the next alignment steps, thus providing the fastest but lowest sensitivity for the seeding step. The values s^{24}_{min} and z_{max} were implemented to filter out alignments that appear unlikely to provide a match and thus reduce processing time (similar to s^{8}_{min}, higher s^{24}_{min} and lower z_{max} values improve speed but reduce sensitivity). We set s^{24}_{min} and z_{max} so that they only filtered out what we considered to be obvious mismatching alignments.

The cutoff score s_{min} can be used to only keep matches that we think are sufficiently well aligned. The s_{min} value was derived using the lowest alignment score s_{lowest} that was observed across all the training alignments (from the last iteration of a paired HMM in the training data). If we were using the training set, then we would set s_{min} such that we only keep alignments that are equal to or better than the lowest score s_{lowest}. In a real alignment scenario however, we may of course have alignments that are correct but with lower scores than s_{lowest}. We therefore used a value for s_{min} that was 10% lower than s_{lowest} (to keep more alignments then a lower s_{min} value should be used). Rounded to the first decimal, this resulted in the same s_{min} value of -2.5 for all of the paired HMMs.

In step 5, given a read length of L_{read} we select a segment of the reference that is also length L_{read} for the alignment. Our paired HMM performs global alignment however, and so there may be indels at the end of the reference or the read which must be considered. We apply rules R1 and R2 for each of these two situations:

R1) If there are gaps at the end of the reference segment then we extend it. For example:

```
A A C G C T - -       A A C G C T A T
A - C G - T A T   →   A - C G - T A T
```

R2) If there are gaps at the end of the read then we remove this last part of the alignment. For example:

```
A - C G - T A T       A - C G - T
A A C G C T - -   →   A A C G C T
```

In the first case, we extend the segment of the reference we are aligning to, and can then simply extend the alignment matrices as required (see Figure 2). In the second case, we assign the aligned sequence the score from the alignment matrix just before the gaps at the end of the read.

Finally, we consider a special case for homopolymers or repeats that can occur for the higher models. For example, we might get the following alignment:

```
T G T - A C C C C C
T G T A A C - C C C
```

If we consider a 2nd order paired HMM, and try to align a C to a C, then the alignment probability C|AC to C|AC is for example different from C|CC to C|CC. If this occurs near the end of an alignment, then a higher order model might not place the gap at the very end of the sequence as shown above. A 0th order model can in contrast be set to always place the gap at the end using the Viterbi algorithm because all the C alignments produce the same probability. To address this issue, we therefore set the higher order models to only use a 0th order model for the last 10 nucleotides of the read when performing a full alignment.

S2 Results

S2.1 Read Mapping - HMMs Trained on Last Alignments

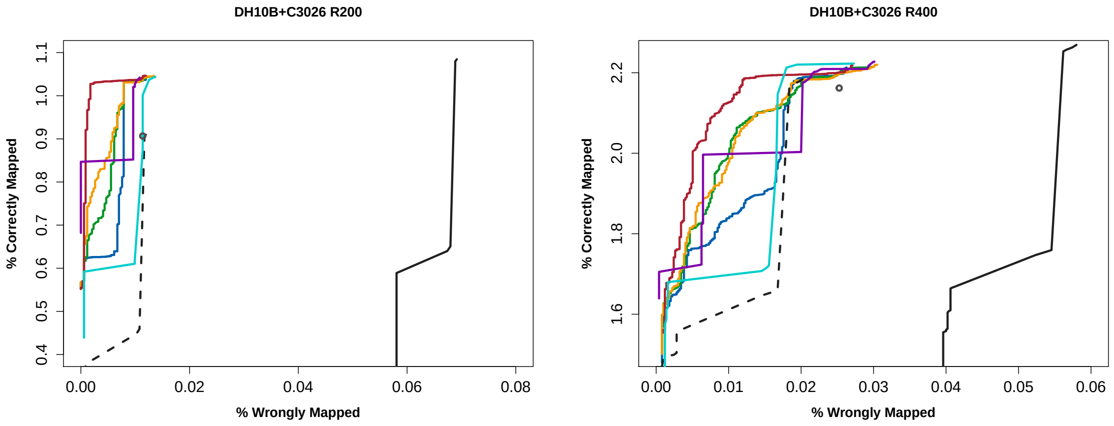

(a) (b)

Figure S1: Comparison of different alignment methods, see Results section in main text for details.

S2.2 Read Mapping - VarHMM Trained on BowTie and TMAP Alignments

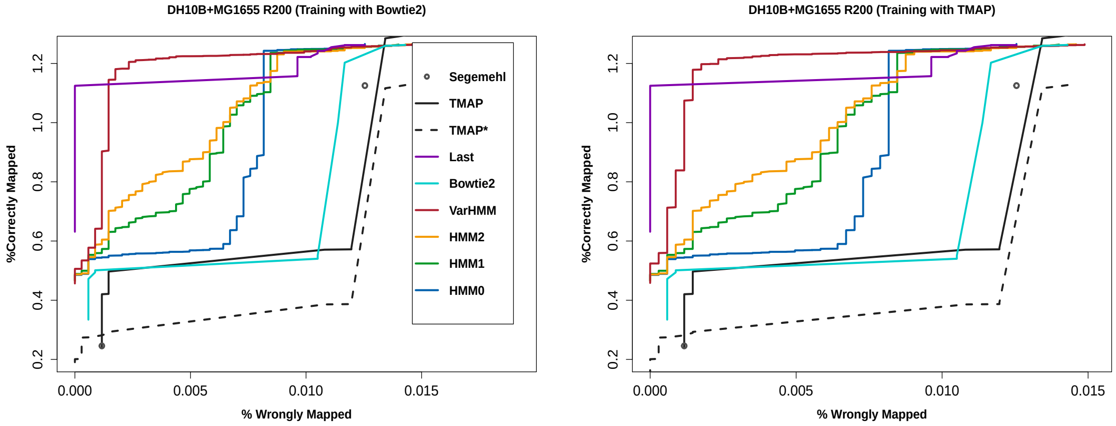

(a) (b)

Figure S2: Comparison of VarHMM with other alignments methods: a) VarHMM trained with a) Bowtie and b) TMAP reads with testing on the R200 dataset to *DH10B* and *MG1655*.

We used Bowtie and TMAP to align the reads from the training dataset described in the Datasets section of the manuscript. We then selected the reads with a score higher than -100 for Bowtie and 50 for TMAP as this yield a similar number of alignments as the Last training dataset. We then trained one VarHMM with the Bowtie alignments and another VarHMM with TMAP alignments as described in the Training section of the manuscript. Alignment testing was performed as in manuscript Figure 4, where we again used the parameters in Table S2 for VarHMM.

S2.3 Read Mapping - c_{min} and Smaller Training Sets

To measure the relationship between c_{min} and the samples in the training data, we used the alignments from Last (that are used for initial parameter estimation as described in the Training section of the manuscript), and calculated the following values for the different orders and states for values of $c_{min} = 50$, $c_{min} = 100$, $c_{min} = 1000$:

Fraction of combinations (FC): The fraction of possible combinations in our aligned training samples that are greater than c_{min}. For example, in the case of the 1st order model and matching state M, we have 256 alignment combinations ($A|A$ to $A|A$, $A|A$ to $A|C$, ...,$T|T$ to $T|T$), and for states X and Y we have 16 possible outputs ($A|A$, $A|C$, ..., $T|T$). If we for example observe AA aligned to AA more than c_{min} times, but all the other alignment possibilities are observed less than c_{min} times in the training data, the FC value would be 1/256 for the 1st order model (and 256/256 if more than c_{min} counts are observed for all possible alignment combinations).

Percent of combinations (PC): FC expressed as a percentage.

Percent of total counts that make up PC (PTC): Sum of total counts for the cases in FC and PC divided by the total counts. For example, if $c_{min} = 100$ and we count 1000 total dinucleotide alignments in the training data, and 950 of these are $A|A$ aligned to $A|A$, then we have only have one case where the count is above 100 (so that $FC = 1/256$), while $PTC = 950/1000 = 95\%$.

The results of our analysis are shown in Table S3. The FC and PC values deteriorated for higher values of c_{min}, while the PTC values only changed a little. This is because mismatches are uncommon, while matching combinations make up a very high percentage of the training alignments, and so for $c_{min} = 1000$ and a 3rd order state M for example, we have $FC = 1024/65536$, while $PTC = 0.99893$. Therefore, when increasing c_{min} or decreasing our training set size, we only exclude more rare cases from the higher order modeling of the M state.

To assess the affects of using a smaller training set and using the same c_{min} value, we trained VarHMM with half of the Last training reads that we used for the VarHMM model seen in the Results section of the manuscript. The results are shown in Figure S3, and as can be seen, the decrease in accuracy was very minor despite using a smaller training set.

$c_{min} = 50$

Order	State	FC	PC	PTC
O1	M	256/256	1.00000	1.00000
	X	16/16	1.00000	1.00000
	Y	16/16	1.00000	1.00000
O2	M	1744/4096	0.42578	0.99997
	X	64/64	1.00000	1.00000
	Y	64/64	1.00000	1.00000
O3	M	4992/65536	0.07617	0.99990
	X	256/256	1.00000	1.00000
	Y	256/256	1.00000	1.00000

$c_{min} = 100$

Order	State	FC	PC	PTC
O1	M	256/256	1.00000	1.00000
	X	16/16	1.00000	1.00000
	Y	16/16	1.00000	1.00000
O2	M	1328/4096	0.32422	0.99997
	X	64/64	1.00000	1.00000
	Y	64/64	1.00000	1.00000
O3	M	3936/65536	0.06005	0.99984
	X	256/256	1.00000	1.00000
	Y	256/256	1.00000	1.00000

$c_{min} = 1000$

Order	State	FC	PC	PTC
O1	M	128/256	0.50000	0.99995
	X	16/16	1.00000	1.00000
	Y	16/16	1.00000	1.00000
O2	M	544/4096	0.13281	0.99975
	X	64/64	1.00000	1.00000
	Y	64/64	1.00000	1.00000
O3	M	1024/65536	0.01563	0.99893
	X	256/256	1.00000	1.00000
	Y	256/256	1.00000	1.00000

Table S3: Training counts and percentages for different values of c_{min}. The first column shows the order (O1 = 1st order, O2 = 2nd order, and O3 = 3rd Order) and the respective states in column 2, while FC, PC, and PTC are shown in the subsequent columns (see text for details).

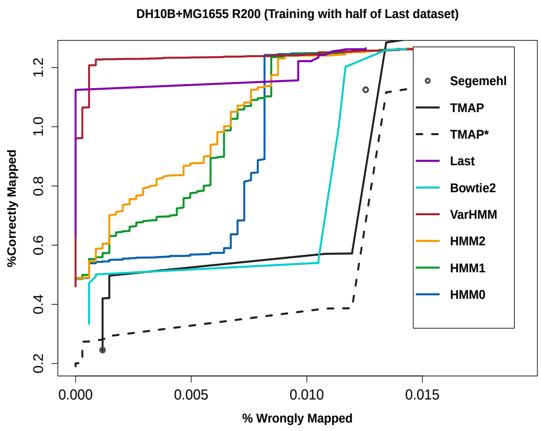

Figure S3: A comparison between VarHMM (trained on half of the Last training dataset) and the other alignment methods, where alignment was made with the R200 dataset to *DH10B* an *MG1655*.

S2.4 Classification

When classifying a pathogen with sequenced DNA reads, high read coverage is often limited to purified samples, and factors such as contaminated samples from other genomic sources or use of multiplexed sequencing can make classification significantly harder (2). Francis *et al.* (2) approximate such effects by using sequenced reads sets with low coverage (0.001X, 0.01X, and 0.1X which yielded sets with 92, 924, and 9237 reads respectively). For each of these sets, they generated 1000 different datasets, each containing reads randomly selected from their full dataset of sequenced reads (containing 92370 reads).

In our analysis, we tested VarHMM, Last, Bowtie, and TMAP with the R200 and R400 datasets on the DH10B and MG1655 test set, to see how the classification compared with the read mapping performance described in the Results section of the manuscript. We only show results for 0.01X and 0.1X coverage (which yielded reads for the R200 set and R400), as 0.001X only provided as low as 19 reads (making accuracy difficult to assess), and all of the aligners could perform 100% accurate classification for 1X datasets. Following the Francis *et al.* test framework, we generated 1000 random datasets using each of the datasets with different coverage (i.e. 1000 datasets randomly selected from the 0.01X set, and 1000 datasets selected from the 0.1X dataset). We then measured the percent of the random datasets that were classified to the correct strain.

Similar to the read alignment test in the manuscript Results section, we varied a threshold and assigned reads. We then selected the strain with the most reads assigned to it from a dataset (this performed better for all aligners than other approaches we tried, such as summing the probabilities for the reads assigned to each strain and selecting the strain with the highest sum of probabilities). A read was assigned to a strain if it only aligned to one of the strains, or if the alignment score to one strain was better than the other strain alignment by the threshold that was used.

The classification results can be seen in Figure S4, where VarHMM showed the best results, followed by Last and then Bowtie and TMAP (Segemehl generally showed a relatively high error rate and is not included in the plots for the sake of visual clarity).

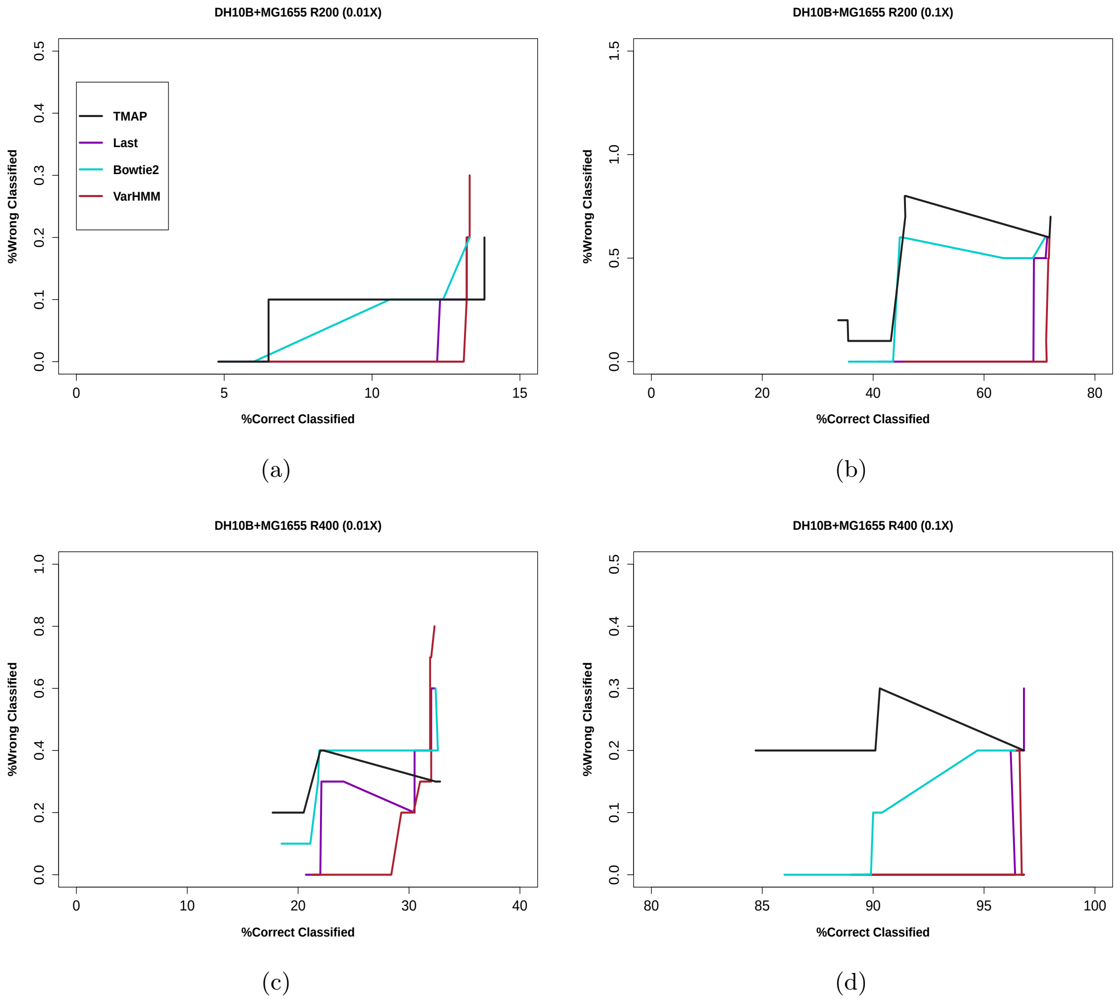

(a) (b) (c) (d)

Figure S4: Classification using different alignment methods. The x-axis shows the % of correctly classified datasets (out of 1000), while the y-axis shows % of wrongly classified datasets (out of 1000). R200 reads with a) 0.01X and b) 0.1X coverage aligned to *DH10B* and *MG1655*, R400 reads with c) 0.01X and d) 0.1X coverage aligned to *DH10B* and *MG1655*.

S3 Aligner Version Details

Version details for the different alignment methods used can be seen in table S4.

Software	Version
Bowtie2	2.2.8
Last	737
Segemehl	0.2.0 − 418
TMAP	5.07

Table S4: Alignment software and corresponding version that was used.

S4 Last Parameters

S4.1 Using Last to Create Training Data

We ran Last with its default parameters to create the training data set that is described in the Training section of the main text, and used the following command:

lastal -Q1 [dbfilename] [fastq filename] | last-map-probs -m1 > [output filename]

S4.2 Training and Alignment

Training with Last was performed using last-train (with default parameters) on the C24-698 set of training reads (see main text), and Last was run with the following parameters for seeding and alignment:

lastdb -uNEAR [db filename] [fasta filename]
lastal -p last_ecoli_scorematrix [dbfilename] [fasta filename] | last-map-probs -m1 > [output filename].

Training Last produced the following output which is contained in the last_ecoli_scorematrix file that is passed to the lastal command above (and contains the gap penalty and alignment score matrices amongst other information):

aligned letter pairs: 74418085.5
deletes: 112271.66996
inserts: 122375.045069
delOpens: 108284.977955
insOpens: 117832.329514
alignments: 304026
mean delete size: 1.03682
mean insert size: 1.03855
delOpenProb: 0.0014448
insOpenProb: 0.00157218
delExtendProb: 0.0355093
insExtendProb: 0.0371213

delExistCost: 295
insExistCost: 291
delExtendCost: 304
insExtendCost: 300

substitution percent identity: 99.9634

count matrix:
18653070.2689 358.995692657 7945.90699230 694.369287905
622.212900026 18313946.1650 285.155023829 3892.52699101
3772.21856607 257.843447923 18653617.7400 616.403254594
718.607801808 7721.74626858 370.955490754 18738908.2300

probability matrix:
0.250758 4.82607e-06 0.000106819 9.33458e-06
8.36456e-06 0.246199 3.83341e-06 5.23282e-05
5.07109e-05 3.46625e-06 0.250765 8.28646e-06
9.66042e-06 0.000103805 4.98685e-06 0.251912

score matrix:
126 -861 -581 -803
-811 128 -882 -644
-648 -891 126 -814
-800 -582 -860 125

#last -a 15
#last -A 15
#last -b 15
#last -B 15
#last -S 1
A C G T
A 6 -43 -29 -40
C -41 6 -44 -32
G -32 -45 6 -41
T -40 -29 -43 6

S5 Hidden Markov Models

S5.1 VarHMM

To download and run VarHMM please perform the following steps:

1. Download VarHMM from: https://github.com/gitvarhmm/varhmm
2. Copy the files to whichever directory is preferred, and then create the executable files by typing *make*
3. Type *varhmm* to view the help menu for aligning reads, and type *varhmmtrain* to view the help menu for training. The trained models that were used for testing in the main text can be found in the files: hmm0, hmm1, hmm2, and hmmvar. Each of these files can be used with the *varhmm* command as described in its help menu.

Please see the included ReadMe file for details on step 2 if needed. The alignments performed in the main text can be made by running the following command for a given HMM model:

```
varhmm [reference filename] [reads filename] [hmm model filename]
```

In the case test sets in the main text, we ran VarHMM with 4 threads. Threading can be performed by using the -T flag and in the case of 4 threads:

```
varhmm [reference filename] [reads filename] [hmm model filename] -T 4
```

Training with VarHMM was performed by using the training file generated with Last (see previous section) and the following commands:

```
varhmmtrain -C [maf filename] [training filename]
varhmmtrain [training filename] [output file] [hmm order]
```

S5.2 Training Results Example

Alignment and transition probabilities from training that were used with VarHMM and the 0th, 1st, and 2nd order models are included with the source code described in the previous section and can be used to make alignments. We have also included an example of the probability and transition matrices for the 0th order HMM below (the values included with the source code are *log* scaled while the values below are the probabilities before applying the *log* function):

Probability matrix:

0.250692	1.15374e-06	2.17826e-05	3.35117e-06
2.98026e-06	0.246385	1.05761e-06	1.91329e-05
1.91329e-05	1.16744e-06	0.250908	3.11775e-06
3.77693e-06	2.20433e-05	9.61376e-07	0.251916

Transition matrix:

0	0.0000000	1.000000	0.00000000	0.000999995
0	0.0383125	0.961688	0.00000000	0.000999995
0	0.0016690	0.996688	0.00164328	0.000999995
0	0.0000000	0.960912	0.03908800	0.000999995

If we look at the diagonal in the probability matrix values, where we can see the probability of matching A with A, C with C, ... , and T with T then the values are very similar to the ones in the Last probability matrix which also uses a 0th order model.

References

[1] Hoare, C. A. Quicksort. The Comp. J. 1962;5(1):10–16.

[2] Francis, OE, Bendall M, Manimaran S, Hong C, Clement NL, Castro-Nallar E, Snell Q, Schaalje GB, Clement MJ, Crandall KA, Johnson WE. Pathoscope: Species identification and strain attribution with unassembled sequencing data. Genome Res. 2013;23(10):1721–1729.
